# Anatomical Tochnolegy as Applied to the Domestic Cat

**Published:** 1883-01

**Authors:** 


### REVIEWS.

Anatomical Technology as Applied to the Domestic Cat: An Introduction to Human, Veterinary and Comparative Anatomy. By Burt G. Wilder, B.S.M.D., Professor of Physiology, Comparative Anatomy and Zoology in Cornell University, and of Physiology in the Medical School of Maine, etc., etc., and Simon H. Gage, B. S., Assistant Professor of Physiology and Lecturer on Microscopical Technology in Cornell University, etc., etc.: New York and Chicago, A. S. Barnes & Co. 1882.

This timely publication must prove a valuable boon to the student of the natural sciences, for it not only supplies a long-felt need in its own special province, but it is replete with general suggestions and directions calculated to be of the highest value to the student of chemistry, botany and zoology, as well as to the student of comparative anatomy. The leading feature of the work is its emphatic plea for method in the prosecution of scientific aims. Each day the field of scientific labor widens and each separate branch continues more and more to overlap the others so that the investigator is bewildered in contemplating the number and variety of the lines of thought he must follow out if he means to be abreast of the scientific problems of the day. Economy of labor and of time is therefore of paramount importance, and of this the authors of the work in question must have been thoroughly convinced, for they give evidence on every page of having worked according to the strictest of methods as students, and they have certainly proved thomselves superior teachers by laying down an admirable set of rules for those who desire to follow in their footsteps. Method is of a two-fold sort, the method which must characterizes our thoughts and intellectual habits, the method which logic inculcates, and the method of practice, which has reference to the distribution of time and the selection, preservation and handling of the tools and material with which we have to work. The authors have dealt lucidly and intelligently with both phases of method. Understanding that our intellectual processes depend altogether on language, that vague, unmeaning terms engender corresponding ideas, they first address themselves to the task of furnishing to the student of anatomy a system of terminology which is at once intelligible, founded on the laws of philology and consequently, a fact of extreme importance, easily remembered. But they had to tear down before they could build up; they had to show reason why the present system of anatomical terminology was radically defective before they could with good grace offer a substitute for it. This, however, was not so difficult to do, for no science has been so hampered with a bizare and barbarous nomenclature as human anatomy. The different regions of the brain have been designated in the most fanciful manner without a particle of reference to function. What, with a *corpus album subrotundum tubercula quadrige-*

*mina*, the *Island of Reil*, the *Pons Varolii* it is no wonder that medical students regard the brain as their special *bete noir*.

But the great defect of anatomical nomenclature hitherto has been its lack of adaptibility to the purposes of comparative anatomy. Thus the terms "posterior" and "anterior," which are met with at every line of Gray's Anatomy, utterly fail to describe corresponding regions in quadrupeds, and so with respect to the majority of regional terms in anatomy.

The authors, therefore, have wisely discarded all terms that are not equally applicable to the cat as well as to the human subject whenever there is question of similar organs. This procedure has the effect at once of greatly simplifying the study of comparative anatomy, and indeed makes a great portion of it a mere reproduction of knowledge previously acquired. Inflection is of the utmost value in the construction of scientific terms, since thereby, through the intervention of a very slight change, the same term may serve to designate a great variety of purposes. The words *ental* and *ectal*, which are of the authors' introduction exhibit an instance of this value of terminal pliancy, since from signifying region they may be made to indicate direction by changing *l* into *d*.

In the chapter on the general description of the skeleton the value of this clear and scientific system of terminology can especially be appreciated. Here the careful student can follow the minutest subdivision and distribution of parts without confusion or uncertainty springing up in his mind to arrest his labors, or what has often happened to lead him into error. The authors have not disdained to give apparently trifling directions in the practical details of dissecting. It is only in appearance that such details are trifling for in reality they are highly important to the uninitiated beginner who thereby is enabled to save much time and labor. Should the student ever flag in his work he need but read over the series of timely and pithy aphorisms selected from the best known writers, and he will be stimulated to renewed exertion and find his enthusiasm kindled afresh. But we have not space to insist at greater length upon the valuable features of this instructive treatise. We can only say, in all sincerity, that it will enable the student to compress an immense deal of work into a very short space of time, and impart a character of more thorough efficiency to his labors.

